# Association between sensitization to common fungi and severe asthma

**DOI:** 10.3389/fpubh.2025.1582643

**Published:** 2025-05-22

**Authors:** Ziqiu Chen, Yu He, Huaien Bu

**Affiliations:** ^1^School of Chinese Materia Medica, Tianjin University of Traditional Chinese Medicine, Tianjin, China; ^2^School of Public Health, Tianjin University of Traditional Chinese Medicine, Tianjin, China

**Keywords:** meta-analysis, sensitization to fungi, fungal genera, *aspergillus* spp., severe asthma

## Abstract

**Background:**

The term severe asthma with fungal sensitization (SAFS) has been coined due to the non-negligible role of fungal sensitization in the development of asthma. These patients typically exhibit poorer lung function, worse clinical prognosis, and a significantly elevated risk of life-threatening asthma exacerbations.

**Methods:**

We conducted electronic searches in three databases as of October 31, 2024. Two evaluators independently screened titles and abstracts to identify studies for full-text review. For studies meeting inclusion and exclusion criteria, the investigators used the JBI Critical Appraisal Checklist and the Newcastle-Ottawa Scale (NOS) to evaluate the quality of cross-sectional studies and a case–control study, respectively, followed by data extraction from included studies.

**Results:**

Among the 10 fungal genera examined, sensitization to *Aspergillus*, *Penicillium*, and *Cladosporium* spp. was significantly associated with an increased risk of severe asthma, with pooled odds ratios (ORs) and 95% confidence intervals (CIs) of 2.36 (1.29–4.31), 1.75 (1.02–3.03), and 2.63 (1.76–3.92), respectively. Within the *Aspergillus* spp., *Aspergillus fumigatus*-specific sensitization demonstrated a stronger association with severe asthma (OR = 2.98, 95% CI: 1.32–6.75). Subgroup analyses further revealed that *Aspergillus* (*A. fumigatus*) sensitization was more strongly linked to severe asthma risk in younger and male populations: ORs with 95% CIs were 2.55 (1.35–4.83) in the ≥40 years subgroup, 3.04 (1.01–9.12) in the <40 years subgroup, and 2.77 (1.16–6.62) in the female-majority subgroup.

**Conclusion:**

In this study, we quantified the risk of sensitization to distinct fungal genera/species, aiming to provide a scientific rationale for screening high-risk fungal sensitization, early detection of severe asthma risk, and personalized health management for patients.

**Systematic review registration:**

https://www.crd.york.ac.uk/PROSPERO/, identifier [CRD42024620737].

## 1 Introduction

Asthma is a complex airway inflammatory disorder characterized by airflow limitation and airway hyperresponsiveness (1). While the majority of asthmatic patients can achieve effective disease control with low-dose medications, approximately 3–8% exhibit severe asthma. These individuals experience significant limitations in daily activities and may even encounter life-threatening clinical events (2).

Dampness and mold make significant contributions to the increase and exacerbation of asthma. One study estimate that 21% of asthma cases in the United States may be attributable to residential dampness and mold (3). Fungi not only harm respiratory health, but also increase socioeconomic burden. A study estimated that the economic burden of asthma onset due to moisture and mold in the United States alone is $15.1 billion (4).

However, the mechanisms of fungal influence on asthma severity are highly complex, involving multiple pathways such as immune modulation (5), antigen release (6), and airway remodeling (7). Fungal sensitization is particularly prevalent in patients with severe asthma (8). This process is primarily mediated by the adaptive immune system, especially through T helper 2 cells (TH2 cells). These cells drive the production of fungal antigen–specific immunoglobulin E (IgE) antibodies involved in allergic and inflammatory response (6). Meanwhile, exposure to fungal allergens and the production of chemokines trigger the recruitment of various immune cells—including eosinophils, neutrophils, and lymphocytes—to the airways. Eosinophils play a crucial role in the inflammatory response to fungal sensitization (7). They release toxic signaling molecules, such as major basic protein, eosinophil peroxidase, and eosinophil cationic protein, which contribute to tissue damage and inflammation. Eosinophils also secrete cytokines and chemokines that regulate asthma severity by interacting with other immune cells (e.g., neutrophils, macrophages, mast cells) (9). Compared with non-sensitized individuals, patients with fungal sensitization have a higher incidence of early-onset asthma and elevated serum interleukin-33 levels (10). Those with severe sensitization tend to have poorer lung function, a poorer prognosis, and a notably increased risk of developing life-threatening asthma exacerbations (11).

Given the role of fungal sensitization in asthma development, the medical community coined the term “Severe Asthma with Fungal Sensitization (SAFS)” to emphasize the important role and clinical significance of fungi in severe asthma (12).

In recent years, there has been a surge in studies on asthma with fungal sensitization. Although prior studies have demonstrated a link between fungal sensitization and severe asthma, many gaps remain in understanding the risks of sensitization to specific fungal genera and species. Epidemiological research has reported a high prevalence of fungal sensitization among asthma patients, with genera such as *Aspergillus* and *Alternaria* frequently implicated. However, extensive quantitative risk assessments for sensitization to these specific fungi are still lacking (13, 14).

Multiple fungal genera have been shown to induce IgE-mediated type I hypersensitivity in susceptible populations. These fungi belong to three main phyla: Ascomycetes, Basidiomycota, and Saprophytes (15). Within the Ascomycota phylum, several genera—including *Aspergillus*, *Penicillium*, *Alternaria*, and *Cladosporium*—are abundant in both indoor and outdoor environments (16) and pose a potential hazard to asthma progression.

Our research focuses on 10 common fungal genera, including *Aspergillus*, *Penicillium*, *Alternaria*, *Cladosporium*, *Helminthosporium*, *Epicoccum*, *Fusarium*, *Candida*, *Mucor*, and *Trichophyton* species, to investigate their contributions to asthma development. Using a rigorous epidemiological approach, we conducted a systematic study. On one hand, we aim to quantitatively evaluate fungal sensitization at the genus level and accurately compare how different fungal sensitizations impact asthma severity; on the other hand, our in-depth analysis of the intrinsic characteristics of patients with *Aspergillus*-triggered severe asthma aids in the investigation of the potential mechanisms and correlates, providing a scientific basis for precision prevention and treatment.

## 2 Methods

### 2.1 Search strategy

We conducted electronic searches in three databases (PUBMED, WEB OF SCIENCE, and EMBASE) on October 31, 2024, following the protocol (PROSPERO reference: CRD42024620737).

To ensure the sensitivity and specificity of the search strategy, our overall search strategy is structured as “severe asthma + fungi + sensitization.” For the concept of “severe asthma,” we employed the terminology recommended by the GINA guidelines (“severe asthma”) alongside multiple synonymous terms (e.g., refractory asthma, difficult-to-control asthma, poorly controlled asthma, irreversible asthma, life-threatening asthma, fatal asthma) to capture relevant literature comprehensively (17). The detailed search strategy is documented in the Supplementary file.

A full search strategy was applied across all three databases to identify eligible articles. We used Endnote version X9 for article management, removing duplicates and selecting studies for full-text screening based on title or abstract; the screening process was documented in a PRISMA flowchart. Two team members independently assessed and screened articles. In cases of disagreement, a third evaluator was consulted, and problems were resolved through discussion.

### 2.2 Eligibility criteria and study selection

The included studies all reported an association between sensitization to common fungal genera and asthma severity.

Inclusion criteria:

- Original peer-reviewed articles reporting primary data;
- Cross-sectional studies, case–control studies, or cohort studies;
- Studies with populations of asthma patients stratified by severity using exact standards and reference guidelines;
- Studies assessing individual sensitization to fungal genera/species, with sensitization diagnostic methods including skin prick tests (SPT) or measurement of specific immunoglobulin E (sIgE) levels;
- Studies reporting and comparing sensitization to fungal genera/species across asthma severity levels (severe vs. non-severe asthma);
- Studies presenting raw data and/or odds ratios (ORs) with confidence intervals (CIs).

Exclusion criteria:

- Duplicated studies or overlapping study populations;
- Studies of the following types: reviews, conference abstracts, meta-analyses, case reports, case studies, and animal experiments;
- Studies lacking required controls (severe vs. non-severe asthma comparison).

### 2.3 Data extraction

The following information was extracted from the studies that meeting the criteria: (1) authors; (2) publication year; (3) publication source; (4) duration of the study; (5) geographic region; (6) study design; (7) sample size; (8) participants’ age, gender, and course of disease; (9) asthma severity classification criteria; (10) diagnostic method of fungal sensitization; (11) counts and/or odds ratios (ORs) with confidence intervals (CIs) for sensitization to fungal genera/species across asthma severity levels.

### 2.4 Quality assessment

The 11 cross-sectional studies and 1 case–control research were evaluated independently by two team members using the JBI Critical Appraisal Checklist and the Newcastle-Ottawa Scale (NOS) (18) (quality assessment of individual studies in the Supplementary file). The JBI scale assigns scores based on the degree of conformity to its items: 0 indicates non-compliance; 1 point signifies mention without detailed description; 2 points denote a complete and comprehensive explanation; an overall score exceeding 70% of the total indicates a low risk of bias. The NOS scale evaluates study quality across three domains—study selection, comparability, and exposure definition—with a maximum score of 9, where higher scores reflect better quality. Two team members independently scored the included literature, and final scores were determined by consensus.

### 2.5 Statistical analysis

All statistical analyses were performed using Review Manager 5.4 software. In this study, we used original data to calculate the unadjusted odds ratios (ORs) and 95% confidence intervals (CIs) for fungal sensitization as an exposure factor between the severe asthma group and the non-severe asthma group (control group). In addition, we directly incorporated ORs from a few studies that reported unadjusted OR values for subsequent calculations.

After completing data collection and organization, we merged data using the random effects model and applied the inverse variance method for weighting. To compute the pooled effect estimates, all ORs and 95% CIs were log-transformed. Specifically, in RevMan’s random-effects model, the Generic Inverse Variance method by default adopts the DerSimonian-Laird estimator. This estimator calculates between-study heterogeneity variance (*Tau^2^*) and dynamically adjusts the weights of individual study effect sizes to reflect the variations among studies, thus achieving a robust estimation of the pooled effect size.

We generated forest plots to directly visualize the study outcomes. In the forest plots, red squares represented the weighted contribution of each study, and *p* < 0.05 was considered statistically significant. The *I*^2^ statistic was used for the outcome test to assess the degree of variation between studies, and heterogeneity was considered to be significant when *I*^2^ was greater than 50%. At last, to ensure the reliability and robustness of the findings, we conducted sensitivity analyses by altering the pooling model and assessed publication bias via funnel plot, respectively.

## 3 Results

### 3.1 Study design characteristics of included studies

**Figure 1:**
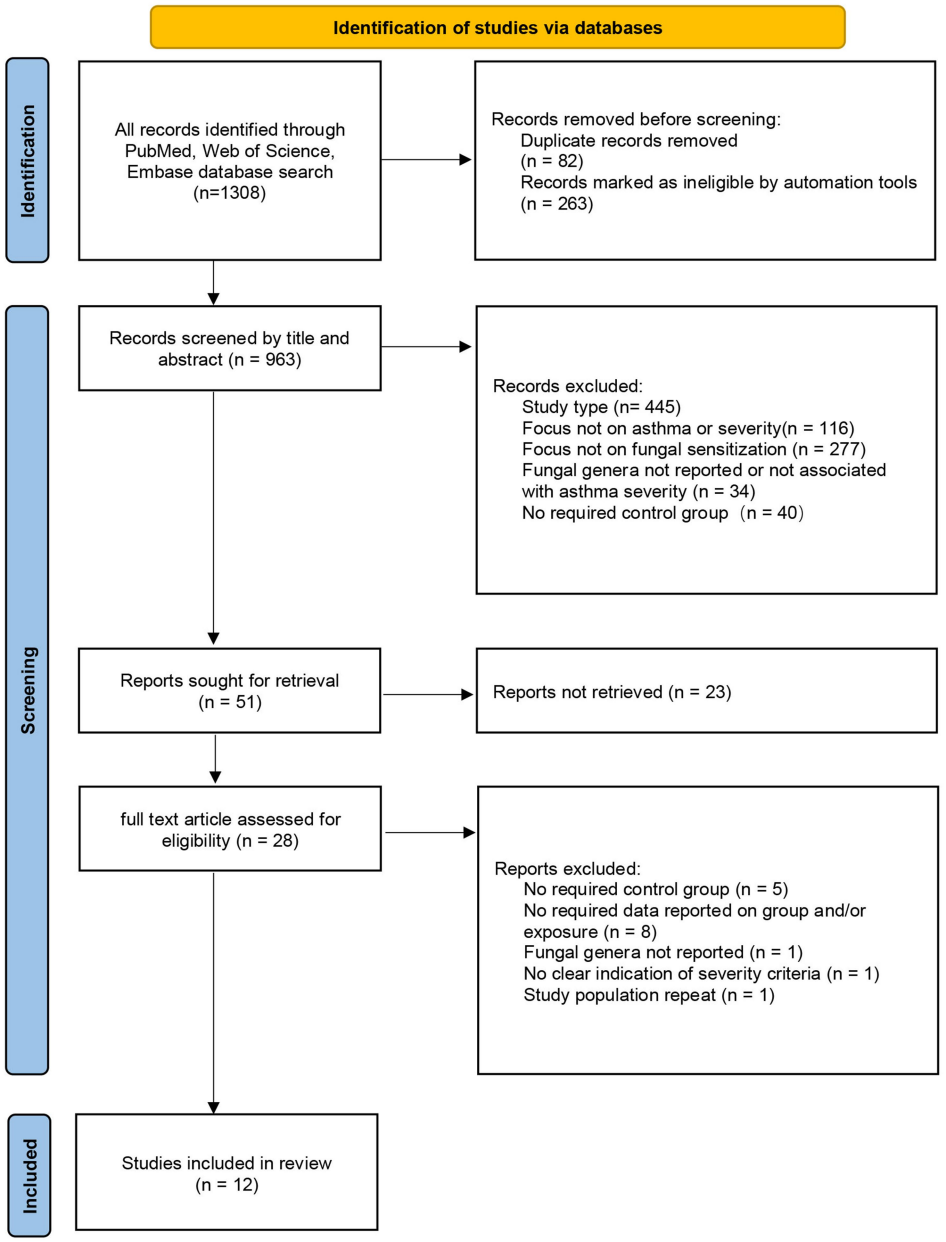
Flowchart of literature screening.

After screening and evaluation, 12 literatures were finally included in this study. Among them, 11 were cross-sectional studies and 1 was a case–control study: Beeh, K. M. 2001 (19), Cazzoletti, L. 2010 (20), Chopra, V. 2017 (21), Gupta, A. 2015 (22), Hayes, D. 2013 (23), Kwizera, R. 2021 (24), Niedoszytko, M. 2007 (25), Ogawa, H. 2011 (26), Saxena, P. 2021 (27), Tanaka, A. 2016 (28), Yeğit, O. O. 2023 (29), Vincent, M. 2018 (30). The flowchart of literature screening is shown in Figure 1. We used the JBI scale and the NOS scale to evaluate the quality of cross-sectional studies and a case–control study, respectively. For the JBI scale, a study was classified as having low bias risk when its score exceeded 70% of the total. Among the 11 studies evaluated with this scale, only one had a score equaling 70%, while the remaining had higher scores. The single study assessed using the NOS scale scored 6 out of 9, indicating moderate quality (detailed in the Supplementary file).

### 3.2 Participant characteristics of included studies

The included studies originated from multiple countries and regions, with geographically diverse settings. Four studies were conducted in Europe: one utilized data from ECRHS II, which included participants from 11 European countries; one was from Germany, one from Poland, and one from Belgium. Of the remaining eight studies, three were from India, two from Japan, and the other three from the United States, Turkey, and Uganda. Based on pooled results (Table 1), all but one study—focusing on children (mean age around10 years)—included participants with a mean/median age over 18 years. Among these, seven studies had participants with a mean/median age under 40, while four reported a mean/median age over 40. Three studies included more male than female participants, and nine had more female than male. Six studies reported age at onset or disease duration.

**Table 1:** Summary of participant characteristics of included studies.

Study	Duration of the study	Country or region	Study design	Study size	Age		Gender (M/F)	
					Severe asthma (Sensitized)	Non-severe asthma (Not sensitized)	Severe asthma (Sensitized)	Non-severe asthma (Not sensitized)
Beeh, K. M. 2001 (19)	Jan. 1995 to Jan. 1996	Frankfurt, Germany	Retrospective investigation	112	42 ± 16	35 ± 15	41/59	44/56
Cazzoletti, L. 2010 (20)	1999 to 2002	25 centers in 11 countries that took part in the ECRHS II	International study	1,127	SPA 45.3 (11.8); MPA 42.6 (13.4)	IA 41.2 (12.8); mPA 41.1 (13.0)	SPA 0.6; MPA 0.77	IA 0.75; mPA 0.59
Chopra, V. 2017 (21)	Jan. 2015 to Dec. 2015	Patiala	Cross-sectional study	282	35.1 ± 13.8 (Sen)	37.1 ± 14.9 (Nsen)	102/104 (Sen)	33/43 (Nsen)
Gupta, A. 2015 (22)	Jul. 2013 to Jun. 2014	Chandigarh, India	Prospective cross-sectional study	100	10.23 (Sen)	10.42 (Nsen)	13/4 (Sen)	66/17 (Nsen)
Hayes, D. 2013 (23)	Aug. 2005 to Dec. 2007	Kentucky	Retrospective cross-sectional study	57	37.9(13.63)^§^		34/72	
Kwizera, R. 2021 (24)	Nov. 2015 to May 2018	Uganda	Cross-sectional study	374	35(26–46)^†^	29(23–41)^†^	68/218	20/68
Niedoszytko, M. 2007 (25)	Not reported	Gdansk, Poland	Cross-sectional study	105	44(19–77); Total 39(18–77)	MA 36(20–59); MiA33(16–59)	13/31; Total 33/72	MA 12/32; MiA 8/9
Ogawa, H. 2011 (26)	Aug. to Oct. 2009	Kanazawa	Reviewed retrospectively	92	64.9 ± 12.1	MA 56.5 ± 15.8; MiA 54.7 ± 15.2	27/19	MA 17/11; MiA 6/12
Saxena, P. 2021^‡^ (27)	Jul. 2017 to Sep. 2018	Chandigarh, India	Prospective observational study	543	39.9(38.0–41.8); Total:36.8(35.6–38.0)	34.9(33.5–36.4)	72/133; Total 224/319	152/186
Tanaka, A. 2016 (28)	Sep. 2014 to Dec. 2014	Tokyo, Japan	Cross-sectional study	160	mean(SD)age was 58.9(15.3)years with a range of 20 to 79 years		73/87	
Vincent, M. 2018 (30)	May 2012 to Sep. 2015	Brussels area	Case–control study	64	55.5 (29.0–66.5) (Sen)	41 (30.0–60.0) (Nsen)	20/12(Sen)	21/11 (Nsen)
Yeğit, O. O. 2023 (29)	Jan. 2022 to Mar. 2023	Istanbul region	Single center retrospective study	77	33.5 (26.0–45.8) (Sen)^§^	33.0 (25.0–45.0) (Nsen)	23/43 (Sen)	28/63 (Nsen)

Course of disease		Asthma severity criteria	Diagnosis of fungal sensitization
Severe asthma (Sensitized)	Non-severe asthma (Not sensitized)		
Not reported		Severe asthma was defined as FEV1 < 60% of predicted value without acute exacerbation at initial presentation (Guidelines for Asthma Diagnosis and Management, 1998).	Total and Specific IgE, SPT
age of onset: SPA 20.0 (25.0); MPA 19.5 (22.0)	mPA 20.0(18.0); IA 17.0 (21.0)	According to the 2002 GINA guidelines, asthma severity was classified by combining clinical severity and subjects’ daily medication regimens.	Specific IgE
Duration: 5.0 (11.5) (Sen)	2.0 (4.5) (Nsen)	Severity of asthma was assessed as per GINA guidelines of 2014	AST with *Aspergillus fumigatus* antigen
Age of onset: 3.5 (Sen)	4.7 (Nsen)	Mild, Moderate and Severe Persistent Asthma as defined by GINA (2006)	SPT, Total Serum IgE level
Not reported		Asthma severity (intermittent, mild, moderate or severe) was assessed per current NAEPP guidelines.	SPT
Not reported		Asthma severity was assessed using a scale based on the National Asthma Education and Prevention Program/National Heart, Lung, and Blood Institute Expert Panel Report (EPR 3) guidelines.	SPT, Specific IgG, Specific IgE, Total IgE
Not reported		Diagnosis and grading of asthma according to Global Asthma Initiative guidelines(2006)	SPT
Not reported		Patients’ asthma severity was categorized per the 2006 GINA guidelines, considering clinical characteristics and lung function.	Intradermal Skin Test, Serological Test IgE
Duration: 12.0(10.6–13.4); Total 10.2(9.4–11.0)	9.02(8.1–10.0)	Asthma severity was assessed according to the 2004 Global Initiative, which consider the treatment’s impact on disease severity.	AST, sIgEAf, sIgGAf, Serum total IgE
age of onset: 32.5(20.9)		Asthma was diagnosed according to the Global Initiative for Asthma (GINA) guidelines(2015)	Serum Total and Allergen Specific IgE Antibodies
Not reported		The GINA criteria (2016) are used to define asthma severity from stage I to V.	SPT, CAP test, Dot-blot assay for mold-specific IgE detection
Not reported		Global Initiative for Asthma (GINA) guidelines for determining asthma severity and treatment steps (2023)	SPT, Serum specific IgE test, Serum total IgE levels

† Only 281 severe asthmatics and 88 non-severe asthmatics had their ages recorded in the study population.

‡ There is an exception: this study defined severe asthma as severe persistent asthma.

§ The population for which age and gender information was recorded in this study includes patients with asthma and allergic rhinitis.

SPA, Severe persistent asthma; MPA, Moderate persistent asthma; mPA, Mild persistent asthma; IA, Intermittent asthma; MA, Moderate asthma; MiA, Mild asthma; Sen, Sensitized; Nsen, Not sensitized; GINA, Global Initiative for Asthma; NAEPP, National Asthma Education and Prevention Program; SPT, Skin prick test; AST, Aspergillus skin test; sIgEAf, *Aspergillus fumigatus* specific IgE; sIgGAf, *Aspergillus fumigatus* specific IgG.

Regarding asthma severity classification, only three of the 12 studies cited external guidelines, while the remaining nine used the Global Initiative for Asthma (GINA) guidelines from various years. Fungal sensitization was diagnosed using skin prick tests (SPT) and measurement of fungal antigen–specific immunoglobulin E (sIgE) levels (6). The diagnostic methods of sensitization corresponding to the sensitization data of the studies we eventually documented were SPT in six studies, sIgE test in three studies, and a combination of SPT and sIgE test in three studies (Table 2).

**Table 2:** Summary table of studies reporting four common indoor and outdoor fungal genera.

Study	Final evaluation method for fungal sensitization	Severe asthma					Non-severe asthma					OR(RR) & CI
		Sensitized				Not sensitized/Total	Sensitized				Not sensitized/Total	
		*Aspergillus* species	*Penicillium species*	*Alternaria* species	*Cladosporium* species		*Aspergillus* species	*Penicillium* species	*Alternaria* species	*Cladosporium* species		
Beeh, K. M. 2001^a^ (19)	Specific IgE and SPT			2		20/22			6		84/90	0.7 (0.1–8.9) (adjusted)
Cazzoletti, L. 2010^a^ (20)	Specific IgE				37	356/393				23	711/734	mPA 5.18 (1.14–23.51); MPA 5.31 (2.54–11.12); SPA 7.22 (2.64–19.79) (adjusted)
Chopra, V. 2017 (21)	AST	*A. fumigatus* 90				3/93	*A. fumigatus* 116				73/189	14.23 (4.23–47.87)
Gupta, A. 2015 (22)	SPT, Total Serum IgE level	*A. flavus* 6; *A. fumigatus* 3; *A. niger* 4; *A. versicolor* 2			3	47/58	*A. flavus* 2; *A. fumigatus* 0; *A. niger* 0; *A. versicolor* 0			1	36/42	Not reported
Hayes, D. 2013 (23)	SPT	1.24 (0.40–4.06) (adjusted); 1.33 (0.45–4.17) (unadjusted)	1.71(0.53–5.98) (adjusted); 1.75 (0.58–5.77) (unadjusted)	1.76 (0.62–5.21) (adjusted); 1.70 (0.61–4.91) (unadjusted)	4.45 (1.29–18.5) adjusted;4.26 (1.30–16.93) (unadjusted)	Total = 32					Total = 25	This study only reports ORs, as mentioned in the previous entry
Kwizera, R. 2021 (24)	SPT	*A. fumigatus* 110				Total = 286	*A. fumigatus* 33				Total = 88	0.94 (0.58–1.52) (unadjusted)
Niedoszytko, M. 2007 (25)	SPT	11	7	9	9	Total = 44	10	7	16	6	Total = 61	Not reported
Ogawa, H. 2011 (26)	Specific IgE	4	4	2		Total = 46	3	2	1		Total = 46	Not reported
Saxena, P. 2021 (27)	SPT	*A. fumigatus* 61				144/205	*A. fumigatus* 103				235/338	Not reported
Tanaka, A. 2016 (28)	Serum Total and Allergen Specific IgE Antibodies	*A. fumigatus* 2.20 (1.10–4.45) (adjusted)	*P. chrysogenum* 1.85 (0.83–4.16) (adjusted)	*A. alternata* 0.92 (0.40–2.08) (adjusted)	*C. herbarum* 1.35 (0.55–3.28) (adjusted)							This study only reports ORs, as mentioned in the previous entry
Vincent, M. 2018 ^a^ (30)	SPT, CAP test, Dot-blot assay for specific IgE detection	*A. fumigatus* 4		*A. alternata* 1		Total = 13	*A. fumigatus* 4		*A. alternata* 6		Total = 51	5.4 (1.00–29.06)
Yeğit, O. O. 2023 (29)	SPT, Serum specific IgE test, Serum total IgE levels	*A. fumigatus* 23				10/33	*A. fumigatus* 10				34/44	Not reported

SPA, Severe persistent asthma; MPA, Moderate persistent asthma; mPA, Mild persistent asthma; IA, Intermittent asthma; MA, Moderate asthma; MiA, Mild asthma; Sen, Sensitized; Nsen, Not sensitized; GINA, Global Initiative for Asthma; NAEPP, National Asthma Education and Prevention Program; SPT, Skin prick test; AST, Aspergillus skin test; sIgEAf, *Aspergillus fumigatus* specific IgE; sIgGAf, *Aspergillus fumigatus* specific IgG. Beeh, K. M. 2001: OR and CI were adjusted for age and sex. Cazzoletti, L. 2010: Adjusted RRRs (95% CIs) for the association between the persistent severity classes and several risk factors. RRRs were also adjusted for type of sample (random vs. symptomatic). RRRs were obtained through a multinomial logistic regression model with the intermittent group as the reference category. Chopra, V. 2017: They conducted multivariate logistic regression analysis to demonstrate that severe asthma is an independent and significant risk factor for AST positivity. Hayes, D. 2013: OR and CI were adjusted for age, sex and race. Kwizera, R. 2021: Due to the fact that the results of specific IgG and IgE tests were not calculated based on severity, only SPT positive individuals were recorded. Ogawa, H. 2011: Due to not all participants participating in SPT, only positive individuals of specific IgE test were recorded. Saxena, P. 2021: Due to the fact that the results of specific IgG and IgE tests were not calculated based on severity, only SPT positive individuals were recorded. Tanaka, A. 2016: OR and CI were adjusted for age and sex. Vincent, M. 2018: This study recorded the “major allergenic fungi.” OR was calculated by comparing patients with severe asthma who were primarily sensitized to *Aspergillus fumigatus* with a control group who were not sensitized to fungi.

a The number of sensitized individuals in these three studies was converted from sensitization rate scores.

### 3.3 Synthesis

We ultimately identified 12 eligible studies meeting the inclusion and exclusion criteria for meta-analysis, examining the association between fungal sensitization—stratified by genus—and severe asthma. Fungi are widely distributed in both indoor and outdoor environments, and they are correlated, albeit species and concentrations differ (31). We focused on the risk of severe asthma due to sensitization to four fungal genera (*Aspergillus*, *Penicillium*, *Cladosporium*, and *Alternaria* species), which are common in both indoor and outdoor environments, and also assessed the risk of six other fungal genera (*Helminthosporium*, *Epicoccum*, *Fusarium*, *Candida*, *Mucor*, and *Trichophyton* species; Tables 2, 3).

**Table 3:** Summary table of studies reporting other fungal genera.

Study	Final evaluation method for fungal sensitization	Severe asthma							
		Sensitized							Total
		*Helminthosporium species*	*Epicoccum* species	*Fusarium* species	*Candida* species	*Mucor species*	*Trichophyton species*	Other	
Gupta, A. 2015 (22)	SPT, Total Serum IgE level	2			*C. albicans 2*	*M. mucedo* 0			58
Hayes, D. 2013 (23)	SPT	1.95 (0.65–6.20) (adjusted); 1.35 (0.48–3.89) (unadjusted)	2.26 (0.65–8.85) (adjusted); 1.57 (0.48–5.68) (unadjusted)	1.39 (0.38–5.53) (adjusted); 0.91 (0.28–3.52) (unadjusted)					32
Niedoszytko, M. 2007 (25)	SPT	13	18	16	25	11		*Trichothecium* 12; *Aureobasidium* 17; *Rhizopus* 11; *Merulius* 10; *yeast* 11; *Chaetomium* 10	44
Ogawa, H. 2011 (26)	Specific IgE				13		5		46
Tanaka, A. 2016 (28)	Serum Total and Allergen Specific IgE Antibodies				*C. albicans* 1.27 (0.66–2.47) (adjusted)	*M. racemosus* 1.23(0.56–2.67) (adjusted)	*T. rubrum* 1.40(0.65–3.01) (adjusted)		

Non-severe asthma								OR(RR) & CI
Sensitized							Total	
*Helminthosporium* species	*Epicoccum* species	*Fusarium* species	*Candida* species	*Mucor* species	*Trichophyton* species	Other		
0			*C. albicans* 0	*M. mucedo* 5			42	Not reported
							25	This study only reports ORs, as mentioned in the previous entry
12	9	18	35	17		*Trichothecium* 13; *Aureobasidium* 11; *Rhizopus* 10; *Merulius* 15; *yeast* 15; *Chaetomium* 12	61	Not reported	
			4		4		46	Positive late skin reaction agains*t S.commune*: 1.82 (1.06–3.24)	
								This study only reports ORs, as mentioned in the previous entry

SPA, Severe persistent asthma; MPA, Moderate persistent asthma; mPA, Mild persistent asthma; IA, Intermittent asthma; MA, Moderate asthma; MiA, Mild asthma; Sen, Sensitized; Nsen, Not sensitized; GINA, Global Initiative for Asthma; NAEPP, National Asthma Education and Prevention Program; SPT, Skin prick test; AST, Aspergillus skin test; sIgEAf, *Aspergillus fumigatus* specific IgE; sIgGAf, *Aspergillus fumigatus* specific IgG. Ogawa, H. 2011: OR was obtained through a multiple logistic regression analysis.

The link between *Aspergillus* spp. and severe asthma has been of great interest (8). We not only quantitatively assessed the strength of this epidemiological association, but also explored the intrinsic characteristics of patients with severe asthma due to *Aspergillus* sensitization through subgroup analyses, with the goal of providing more comprehensive and in-depth insights into this field of study.

Historically, asthma severity has been classified into two major systems. The current standard classification defines severity based on treatment intensity required for control: mild (MiA), moderate (MA), and severe asthma (SA) (17). By contrast, earlier guidelines categorized severity into four tiers—intermittent (IA), mild persistent (mPA), moderate persistent (MPA), severe persistent (SPA)—based on symptom severity, airflow limitation, and lung function variability (32).

For the latter classification criteria, we defined severe asthma in principle as moderate persistent and severe persistent asthma (Table 1). Given the variability in reported data across studies, we derived unadjusted odds ratios (ORs) from raw data or extracted them directly from the literature for subsequent pooled effect estimates.

### 3.4 Results of four common indoor and outdoor fungal genera

**Figure 2:**
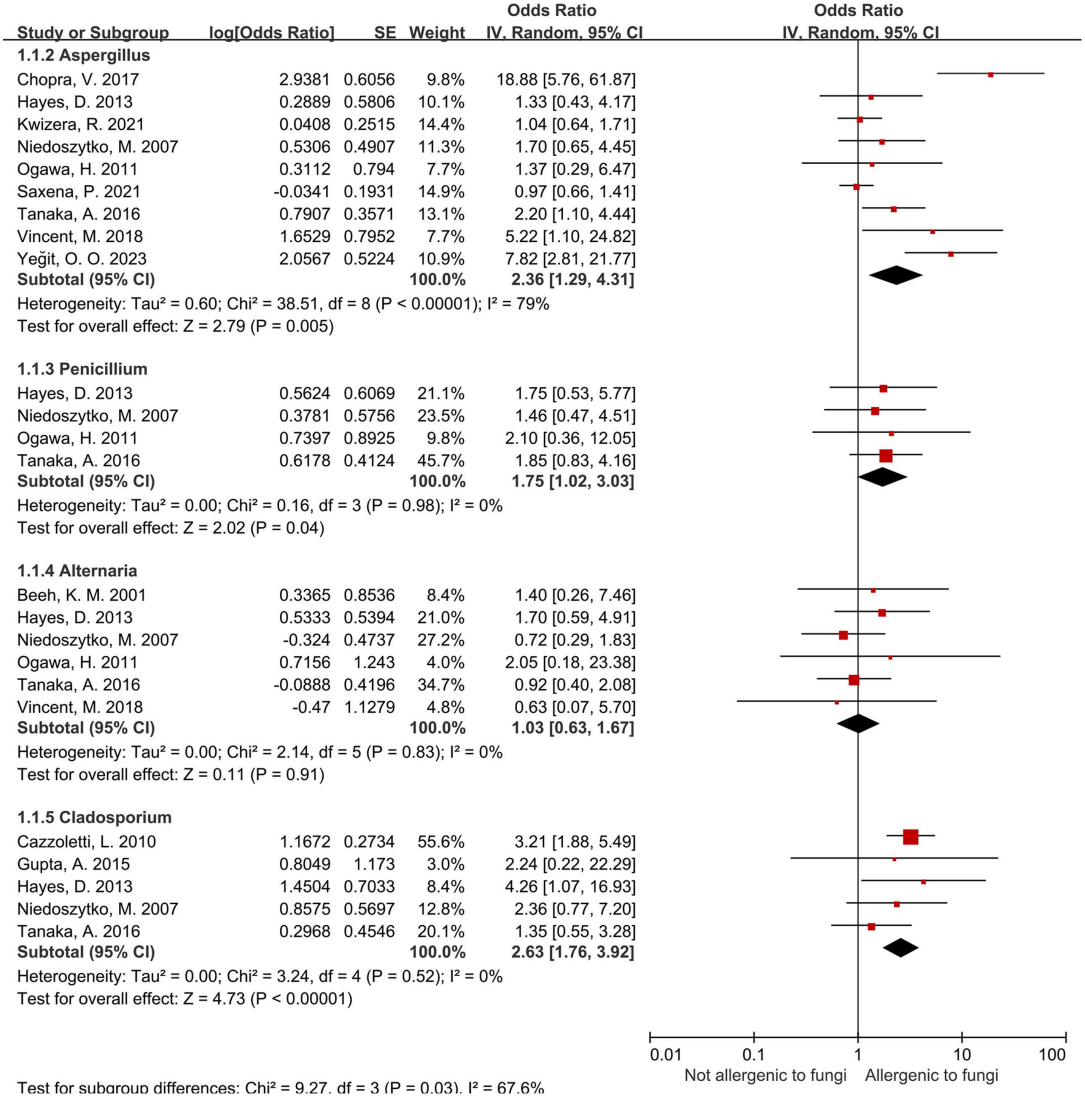
Four most common fungal genera.

Based on the pooled effects of the four most prevalent indoor and outdoor fungal genera, the results of the three subgroups of *Aspergillus*, *Penicillium*, and *Cladosporium* spp. were statistically significant (Table 4; Figure 2). Sensitization to *Aspergillus* spp. was associated with a 2.36-fold increased risk of severe asthma (95% CI: 1.29–4.31); sensitization to *Penicillium* spp. had an OR of 1.75 (1.02–3.03); and sensitization to *Cladosporium* spp. showed an OR of 2.63 (1.76–3.92). Heterogeneity assessed via *I*^2^ indicated high variability among *Aspergillus*-related studies (*I*^2^ = 79%), whereas the remaining genera—including *Alternaria* spp., which showed no significant association—had *I*^2^ values of 0%, indicating low heterogeneity.

**Table 4:** Summary effect estimates.

Model in subgroup analysis	Results synthesis: Severe asthma		
	Number of studies included in analysis	Pooled effect size (95%CI)	*I^2^* value
Model 1: *Aspergillus* species^a^	10	2.36 (1.29,4.31)	79%
Model 2: *Penicillium* species	4	1.75 (1.02,3.03)	0%
Model 3: *Alternaria* species	6	1.03 (0.63,1.67)	0%
Model 4: *Cladosporium* species	5	2.63 (1.76,3.92)	0%
Model 5: *Helminthosporium* species	3	1.62 (0.83,3.16)	0%
Model 6: *Epicoccum* species	2	1.52 (0.68,3.37)	0%
Model 7: *Fusarium* species	2	1.22 (0.61,2.47)	0%
Model 8: *Candida* species	4	1.52 (0.82,2.81)	31%
Model 9: *Mucor* species	3	0.80 (0.32,2.04)	50%
Model 10: *Trichophyton* species	2	1.37 (0.70,2.68)	0%

a Calculation of the pooled effects of Aspergillus genus excluded the study of Gupta, A. 2015, which reported data of four Aspergillus species and were unable to merge. Since two of the original studies (Hayes, D. 2013 and Tanaka, A. 2016) reported only ORs with 95% CIs, we applied generic inverse variance to calculate the pooled effect size, and ORs were calculated by us for the remaining studies which reported raw data. Unadjusted ORs with CIs were selected to calculate the pooled effect size with one exception, Tanaka, A. only reported adjusted ORs with CIs.

### 3.5 Results of six other fungal genera

**Figure 3:**
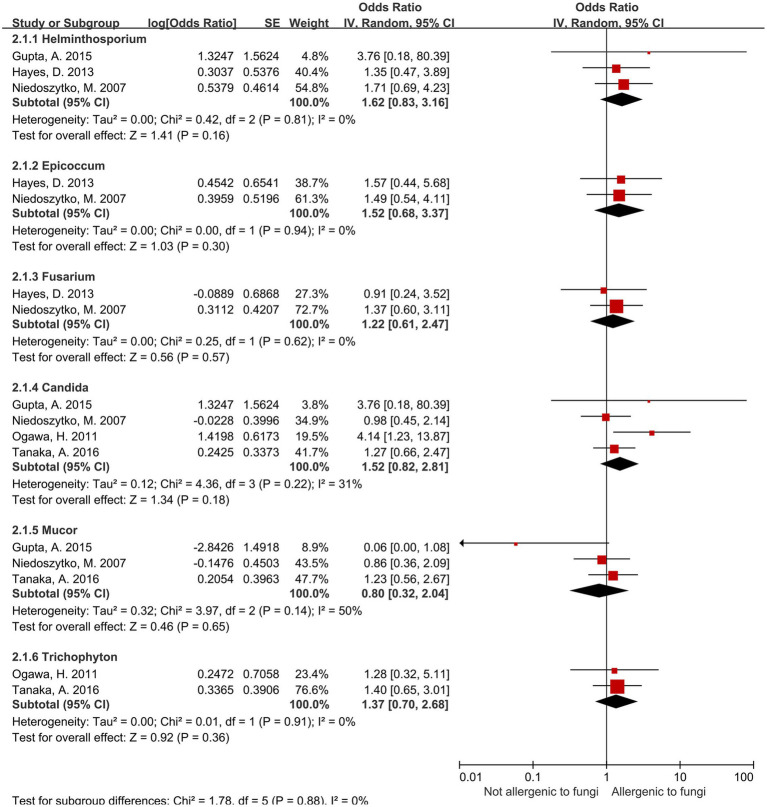
Other six fungal genera.

For the present being, no significant association was observed between sensitization to the remaining six fungal genera—including *Candida* spp.—and increased risk of severe asthma (Figure 3). Heterogeneity assessed via *I*^2^ showed four genera had *I*^2^ values of 0%, while the other two—*Candida* spp. (*I*^2^ = 31%) and *Mucor* spp. (*I*^2^ = 50%)—exhibited moderate heterogeneity.

### 3.6 Stratification analysis of the *aspergillus* group

**Figure 4:**
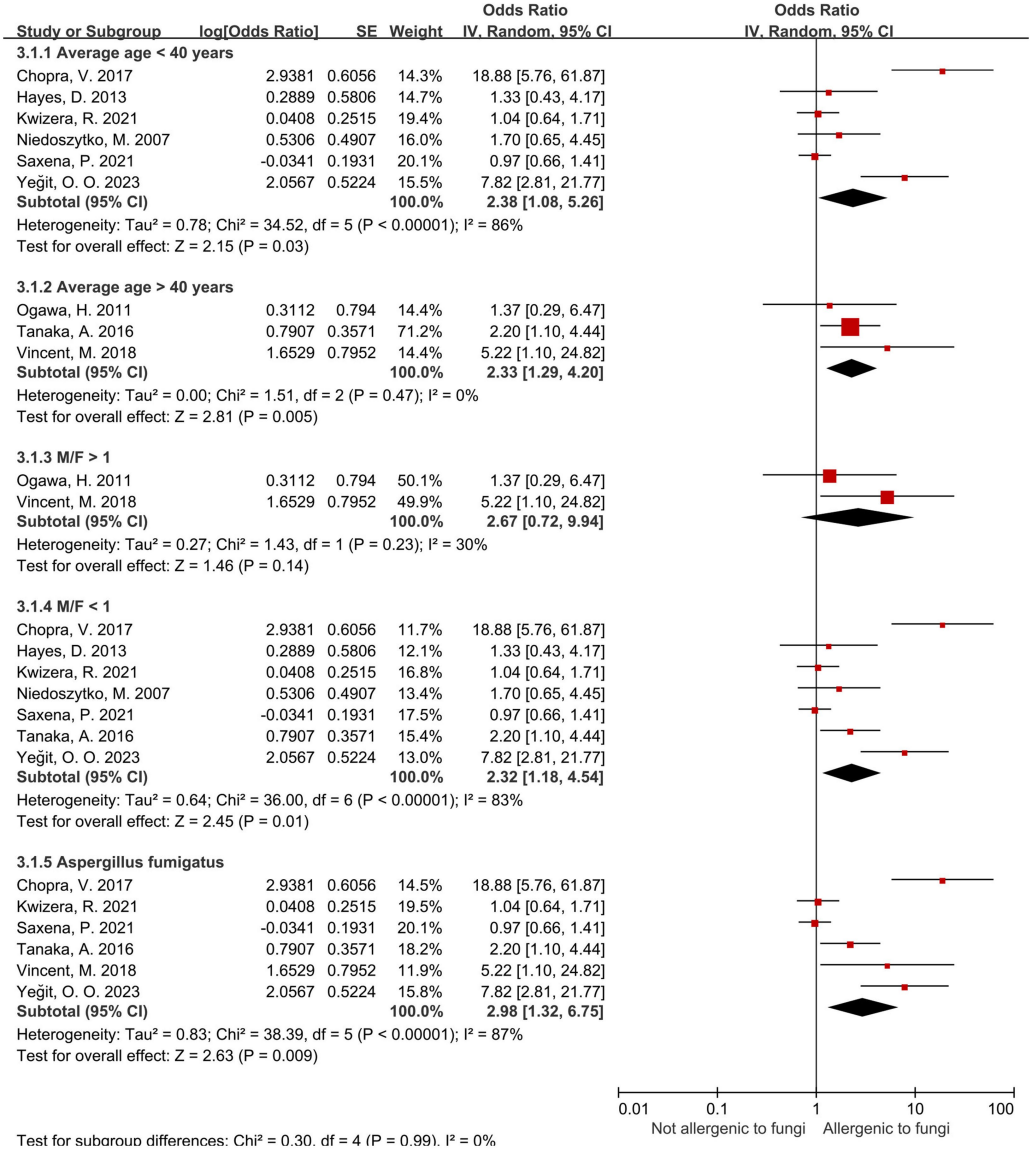
Subgroup analysis of Aspergillus spp. in terms of sex, age, and *Aspergillus fumigatus*.

**Figure 5:**
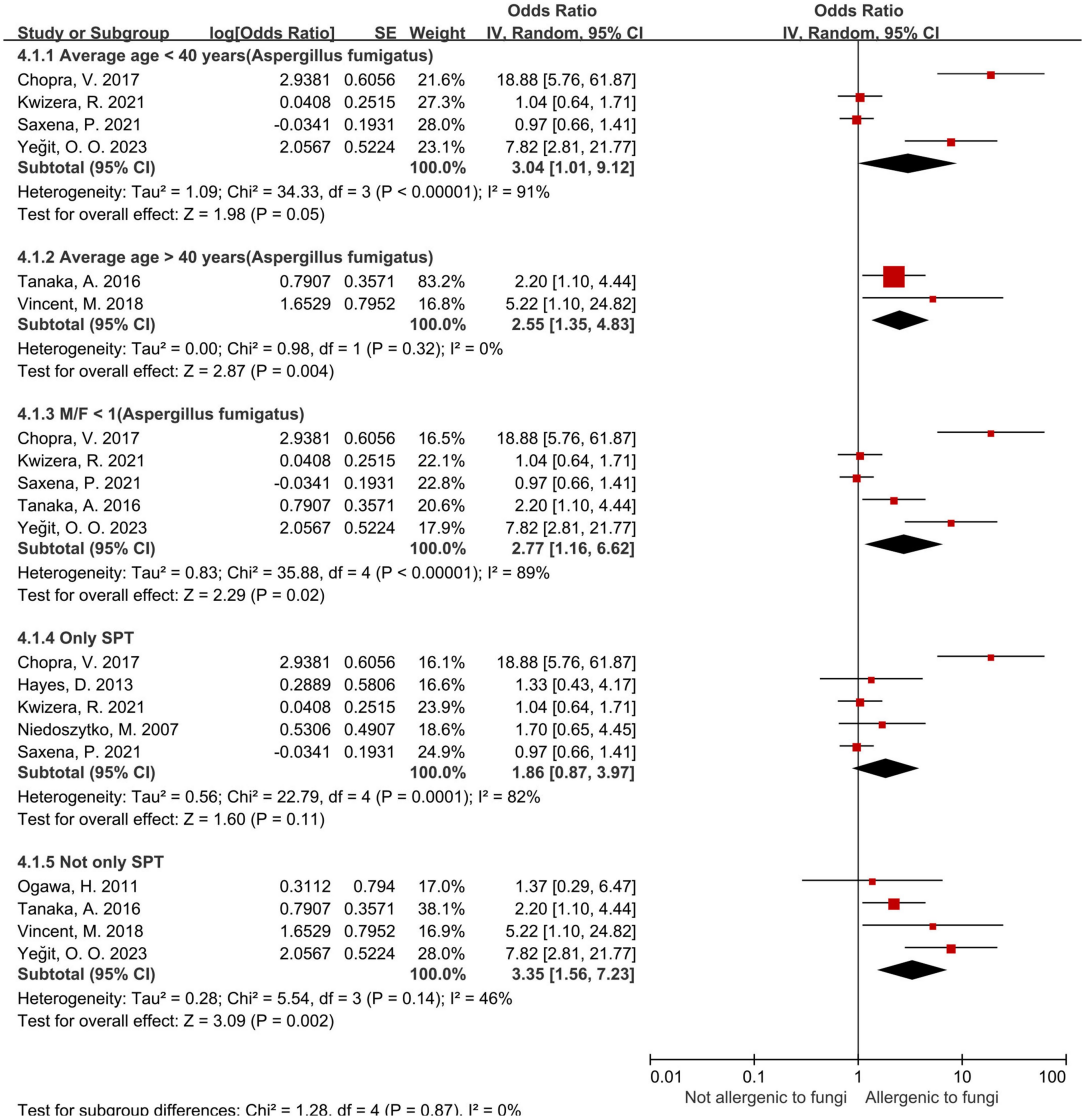
Subgroup analysis of Aspergillus spp. in terms of sensitization diagnosis and sex, age under *Aspergillus fumigatus*.

Regarding the *Aspergillus* spp. group, we conduct subgroup analyses (Table 5) from the perspectives of age, male-to-female ratio, *Aspergillus fumigatus*, age and sex under *Aspergillus fumigatus*, and diagnostic modality of sensitization (Figures 4, 5). In the age-stratified analysis, the OR for the ≥ 40 years group decreased slightly compared to the overall OR, while the <40 years group showed a marginal increase; however, neither change reached statistical significance. By gender, the OR was slightly reduced in female-dominated subgroups, whereas male-dominated subgroups showed no significant association, likely owing to limited sample size. Notably, *Aspergillus fumigatus*-specific sensitization demonstrated a stronger association with severe asthma (OR = 2.98, 95% CI: 1.32–6.75) compared to the broader *Aspergillus* spp. analysis. Following that, we examined the *Aspergillus fumigatus* sensitization group in terms of age and gender individually. The findings for the age-sex subgroup of *Aspergillus fumigatus* were the same as before (age-sex subgroup of *Aspergillus* spp.), but with more significant changes: the ORs with 95% CIs were 2.55 (1.35–4.83) for ≥40 years, 3.04 (1.01–9.12) for <40 years, and 2.77 (1.16–6.62) for female-dominated groups.

**Table 5:** Heterogeneity analysis of *Aspergillus* species.

Model in subgroup analysis	Results synthesis: Severe asthma		
	Number of studies included in analysis	Pooled effect size (95%CI)	*I^2^* value
Model 1: Average age < 40 years	6	2.38 (1.08,5.26)	86%
Model 2: Average age > 40 years	3	2.33 (1.29,4.20)	0%
Model 3: M/*F* > 1	2	2.67 (0.72,9.94)	30%
Model 4: M/*F* < 1	7	2.32 (1.18,4.54)	83%
Model 5: *Aspergillus fumigatus*	6	2.98 (1.32,6.75)	87%
Model 6: Average age < 40 years (*Aspergillus fumigatus*)	4	3.04 (1.01,9.12)	91%
Model 7: Average age > 40 years (*Aspergillus fumigatus*)	2	2.55 (1.35,4.83)	0%
Model 8: M/F < 1 (*Aspergillus fumigatus*)	5	2.77 (1.16,6.62)	89%
Model 9: Only diagnose with SPT	5	1.86 (0.87,3.97)	82%
Model 10: Not only diagnose with SPT	4	3.35 (1.56,7.23)	46%

### 3.7 Risk of bias of individual studies

We evaluated included cross-sectional and case–control studies with the JBI and NOS scales, respectively (detailed in the Supplementary file), finding generally low bias and comparable quality across studies. Since we ultimately calculated the unadjusted odds ratio (OR) without controlling for confounding factors, there is potential for bias in the results. Additionally, the study by Tanaka (28) only presented the adjusted OR, and we included it in our calculation process. This approach may also lead to the occurrence of bias. Funnel plots were used to assess publication bias for five fungal genera (*Aspergillus*, *Penicillium*, *Cladosporium*, *Alternaria*, and *Candida* spp.), as fewer than four studies existed for the remaining genera, precluding funnel plot analysis. The funnel plot revealed some publication bias in studies of *Aspergillus*, *Alternaria*, and *Candida* spp. (in the Supplementary file). Moreover, sensitivity analyses, conducted by changing the data pooling model, showed consistent conclusions in nature between fixed- and random-effects models, suggesting the robustness of our findings.

## 4 Discussion

During the evaluation of the studies on fungal sensitization associated with severe asthma, we carried out a structured systematic evaluation in strict accordance with a pre-published protocol. Our meta-analysis of the association between sensitization to specific fungal genera and severe asthma revealed that, of the 10 fungal genera involved in the study, sensitization to *Aspergillus*, *Penicillium*, and *Cladosporium* spp. significantly increased the risk of severe asthma, among which sensitization to *Aspergillus* and *Cladosporium* spp. conferred relatively higher risks. When comparing the ORs, sensitization to *Cladosporium* spp. was no less essential for the risk of severe asthma than the more widely studied sensitization to *Aspergillus* spp.; within the *Aspergillus* spp., *Aspergillus fumigatus* showed a particularly strong association with increased risk. Subgroup analyses further highlighted population-specific patterns, such as *Aspergillus* (*A. fumigatus*) sensitization being more strongly linked to severe asthma risk in younger and male populations. In addition to the primary conclusions listed above, the included studies yielded some intriguing observations. Cazzoletti (20) reported a dose–response relationship: increasing asthma severity correlated with higher *Aspergillus* sensitization, reinforcing the likelihood of a causal link (Table 2). This finding implied that effective control of fungal sensitization is important in asthma management. The study by Ogawa (26) identified another fungus that may enhance the risk of severe asthma after sensitization (*S. commune*: 1.82 [1.06–3.24]; Table 3).

Sensitization to fungi is significantly associated with severe asthma, which frequently provokes undesirable clinical consequences such as increased ICU admissions and mechanical ventilation requirements (11). However, the effects of different fungal allergens on asthma vary, highlighting notable species-specific impacts (33). In this study, we quantified the risk magnitude of sensitization to distinct fungal genera/species, aiming to provide a theoretical foundation for both screening high-risk fungal sensitization and early identification of severe asthma risk. Secondly, for patients with poorly controlled or severe asthma, this study points in the direction of optimizing asthma management and developing targeted interventions, with the expectation of informing clinical practice and enhancing patient outcomes and quality of life (34). Current therapeutic approaches for severe asthma with confirmed fungal sensitization have advanced into clinical research and application phases. Antifungal treatments, such as Itraconazole (35) and Omalizumab (36), have demonstrated efficacy in improving disease control for these patients. Biological therapies targeting eosinophilic inflammation, including Mepolizumab or Benralizumab (37), have also shown clinical promise. In parallel with the above implications for clinical practice, the study proposes tailored disease management strategies and patient-centered recommendations to enhance quality of life through personalized self-health management. *Aspergillus*, *Penicillium*, and *Cladosporium* spp. are common fungi in both indoor and outdoor environments (16), with their concentrations influenced by environmental (38), seasonal, and humidity conditions. Fungi proliferate more rapidly in autumn (39) and winter (40), as well as in humid (41, 42) conditions, leading to increased production of spores, mycelium, and fragments (43), also increase. The fungi listed above are not only ubiquitously present in air but also deeply intertwined with human activities. *Aspergillus oryzae* and *Aspergillus niger* are widely used in food fermentation and the industrial production of organic acids (citric acid) (44); *Cladosporium* can produce a variety of beneficial enzymes, which play an important role in the field of microbial chemistry and plant science (45); *Penicillium chrysogenum* serves as a key penicillin-producing strain and synthesizes bioactive secondary metabolites, which show excellent biosynthesis potential (46). For workers in the above industries, exposure to bioaerosols, including fungal spores, represents a significant occupational hazard that cannot be ignored. Therefore, in order to prevent the asthma deterioration, asthmatics may need to minimize exposure to humid environments and avoid activities with high-risk fungal contact.

This study had several limitations. On the one hand, the number of included studies was relatively small and the evidence grade low (11 cross-sectional studies and 1 case–control study), which somewhat obscured potential associations between sensitization to other fungal genera and severe asthma and limited demographic-based stratification. Moreover, after the calculation and combination of the data, what we eventually obtained is the unadjusted odds ratio (OR). This methodology presents a risk of result bias, which is consistent with that observed in other studies of a similar nature. On the other hand, the analysis of *I*^2^ revealed high heterogeneity among the nine *Aspergillus* spp.-related studies. In order to explore the source of heterogeneity and to investigate the intrinsic characteristics of patients with *Aspergillus*-sensitized severe asthma, we conducted subgroup analyses across dimensions such as age and gender. Sensitization diagnostic methods provided clues to heterogeneity (Table 5). Studies using only skin prick tests (SPT) showed high heterogeneity and non-significant pooled effects, whereas subgroups using sIgE tests (alone or combined with SPT) had significantly lower heterogeneity. SPT and the sIgE test are both fundamental diagnostic methods for sensitization, each with distinct advantages and limitations that make them non-interchangeable (47). SPT is often influenced by the quality of the reagent and the experience of the operator, which may explain the large heterogeneity among studies that used SPT alone to diagnose *Aspergillus* sensitization. Although subgroup analyses revealed some intrinsic characteristics of disease groups, such as sensitization to *Aspergillus* spp. (*Aspergillus fumigatus*) being more likely to pose a risk of severe asthma in younger age groups and in the male population, unresolved heterogeneity hinders definitive conclusions. Besides, it is also worth pointing out that potential covariates were not considered in this study. For example, other allergens such as dust mites also contribute to asthma exacerbation, for which the effects of these factors on the strength and direction of the association between sensitization to distinct fungal genera/species and severe asthma remain unclear. Looking ahead, more comprehensive diagnostic approaches for sensitization (e.g., SPT combined with sIgE tests), large-sample multicenter studies, and more robust evidence of causality may help us to uncover more quantitative associations between sensitization to identified genera/species of fungi and severe asthma, as well as their specific impact on diverse populations.

Fungal sensitization is a key mechanism underlying the increased risk of severe asthma following fungal exposure. However, the effects of fungal exposure on respiratory health are complex and multifaceted, with distinct fungal communities at varying concentrations exerting differential impacts (48). It has even been proposed that exposure to species-rich fungal environments may inversely reduce asthma risk (49). As molecular biology advances, our understanding of fungal sensitization mechanisms in asthma deepens, yet multiple perspectives are still needed to precisely clarify how fungi contribute to asthma development and severity exacerbation. This requires deeper insights into the role of fungal metabolites and fragments in these processes, as well as the detailed immunoinflammatory response mechanisms (50, 51). It is of great significance to investigate these mechanisms. On the one hand, it can fulfill the gaps in our knowledge about fungi’s biological features; on the other hand, it can pave the path for the development of targeted therapies and bring new therapeutic hope to asthma patients. Given fungi’s pivotal role in asthma, it will undoubtedly become a key research direction in the future to elucidate the roles and underlying mechanisms of biologics and antifungal therapies in the management of fungal sensitization in asthma through animal experiments as well as randomized clinical trials (6). Furthermore, emerging evidence suggests genetic susceptibility may influence individual vulnerability to fungal-associated asthma (52, 53). Nevertheless, this area remains poorly understood, and more in-depth studies are urgently needed to fully characterize the gene–environment interactions driving fungal-associated asthma. We anticipate that more extensive studies will be conducted in the future to further explore and examine the underlying mechanisms, clarify the role of relevant genetic factors, develop targeted therapies, and implement effective controls to improve patient outcomes.

## Data Availability

The original contributions presented in the study are included in the article/Supplementary material, further inquiries can be directed to the corresponding author.
