# Supplementary material for: Association between sensitization to common fungi and severe asthma
Source: Front Public Health. 2025 May 22;13:1582643. doi: 10.3389/fpubh.2025.1582643 (PMC12139209; doi:10.3389/fpubh.2025.1582643)
Supplement: Supplementary file 1 [file Data_Sheet_1.docx]

Supplementary Material

1. Search strategy

severe asthma + fungi +sensitization

(((((((("severe asthma") OR ("refractory asthma")) OR ("difficult to control asthma")) OR ("poorly controlled asthma")) OR ("irreversible asthma")) OR ("life-threatening asthma")) OR ("fatal asthma")) AND ((((((((Hypersensitivities[MeSH Terms]) OR (Allergy[Title/Abstract])) OR (Allergies[Title/Abstract])) OR (Allergic Reaction[Title/Abstract])) OR (Allergic Reactions[Title/Abstract])) OR (Reaction, Allergic[Title/Abstract])) OR (Reactions, Allergic[Title/Abstract])) OR (Sensitization[Title/Abstract]))) AND ((((((((Fungi[MeSH Terms]) OR (fungus[Title/Abstract])) OR (Fungi, Filamentous[Title/Abstract])) OR (Filamentous Fungi[Title/Abstract])) OR (Filamentous Fungus[Title/Abstract])) OR (Fungus, Filamentous[Title/Abstract])) OR (Molds[Title/Abstract])) OR (Mold[Title/Abstract]))

1. Supplementary Figures and Tables
   1. Table S1. Quality Assessment of a case-control study included using the Newcastle-Ottawa Scale

| **Studies** | **Selection** | | | | **Comparability** | **Exposure** | | | **Final quality score** |
| --- | --- | --- | --- | --- | --- | --- | --- | --- | --- |
|  | 1 | 2 | 3 | 4 | 1 | 1 | 2 | 3 |  |
| Vincent, M. 2018 |  |  |  |  |  |  |  |  | 6/9 |
| All entries are worth a maximum of 1 point out of 9, except for comparability, which is worth a maximum of 2 points. | | | | | | | | | |

- 1. Table S2: Quality Assessment of cross-sectional studies included using the JBI Critical Appraisal Checklist

| **Studies** | **Purpose of the study and rationale for the topic** | **Selection of the study population** | **Inclusion and exclusion criteria** | **Description of sample characteristics** | **Tools for data collection** | **Measures to verify information** | **Ethical issues** | **Statistical method** | **Presentation of the results** | **Value of the study** | **Final quality score** |
| --- | --- | --- | --- | --- | --- | --- | --- | --- | --- | --- | --- |
| Beeh, K. M. 2001 | 2 | 2 | 2 | 2 | 2 | 0 | 0 | 2 | 2 | 2 | 16/20 |
| Cazzoletti, L. 2010 | 2 | 2 | 2 | 2 | 2 | 1 | 1 | 2 | 2 | 2 | 18/20 |
| Chopra, V. 2017 | 2 | 1 | 2 | 2 | 2 | 0 | 2 | 2 | 2 | 2 | 17/20 |
| Gupta, A. 2015 | 2 | 1 | 2 | 2 | 2 | 0 | 2 | 1 | 2 | 2 | 16/20 |
| Hayes, D. 2013 | 2 | 1 | 1 | 1 | 2 | 0 | 1 | 2 | 2 | 2 | 14/20 |
| Kwizera, R. 2021 | 2 | 2 | 2 | 2 | 2 | 0 | 2 | 2 | 2 | 2 | 18/20 |
| Niedoszytko, M. 2007 | 2 | 1 | 1 | 1 | 2 | 0 | 2 | 2 | 2 | 2 | 15/20 |
| Ogawa, H. 2011 | 2 | 1 | 1 | 2 | 2 | 0 | 2 | 2 | 2 | 2 | 16/20 |
| Saxena, P. 2021 | 2 | 2 | 2 | 2 | 2 | 0 | 2 | 2 | 2 | 2 | 18/20 |
| Tanaka, A. 2016 | 2 | 1 | 1 | 2 | 2 | 0 | 2 | 2 | 2 | 2 | 16/20 |
| Yeğit, O. O. 2023 | 2 | 1 | 1 | 2 | 2 | 0 | 2 | 2 | 2 | 2 | 16/20 |
| Scores were given according to the degree of compliance with the criteria: 0 for no compliance; 1 for mentioned but not described in detail; and 2 for detailed and comprehensive description. A general score > 70 percent of the total score is considered to be a low risk of bias. | | | | | | | | | | | |

- 1. Table S3: The calculation process of ten common indoor and outdoor fungal genera

We extracted the original data (the numbers of individuals with and without sensitization to different fungal genera among populations with severe asthma and those with non-severe asthma) and the odds ratio (OR) values from the included studies. Using the built-in calculator of RevMan, we calculated some of the OR values with the original data. Then, we listed these calculated OR values together with the OR values directly reported in the literatures in Excel according to the fungal genera for subsequent combined calculations.

After completing the data collection and organization, we combined the data using a random-effects model. We performed logarithmic transformation on all OR values and their 95% confidence intervals (CIs) through the built-in calculator of RevMan, and applied the inverse variance method for weighting. The Generic Inverse Variance method by default employs the DerSimonian-Laird estimator. This estimator calculates the between-study heterogeneity variance (*Tau²*) and dynamically adjusts the weights of the effect sizes of individual studies to reflect the differences among studies, thus achieving a robust estimation of the pooled effect size.

| ***Aspergillus* species** | **Sensitized / total** | | **OR and 95% CI** | **log [Odds Ratio]** | **SE** | **Weight** |
| --- | --- | --- | --- | --- | --- | --- |
|  | **Severe asthma** | **Non-severe asthma** |  |  |  |  |
| Vincent, M. 2018 | 4/13 | 4/51 | 5.22 [1.10, 24.82] | 1.6529 | 0.7952 | 7.7% |
| Ogawa, H. 2011 | 4/46 | 3/46 | 1.37 [0.29, 6.47] | 0.3112 | 0.794 | 7.7% |
| Chopra, V. 2017 | 90/93 | 116/189 | 18.88 [5.76, 61.87] | 2.9381 | 0.6056 | 9.8% |
| Hayes, D. 2013 | Not reported | Not reported | 1.33 [0.43, 4.17] | 0.2889 | 0.5806 | 10.1% |
| Yeğit, O. O. 2023 | 23/33 | 10/44 | 7.82 [2.81, 21.77] | 2.0567 | 0.5224 | 10.9% |
| Niedoszytko, M. 2007 | 11/44 | 10/61 | 1.70 [0.65, 4.45] | 0.5306 | 0.4907 | 11.3% |
| Tanaka, A. 2016 | Not reported | Not reported | 2.20 [1.10, 4.44] | 0.7907 | 0.3571 | 13.1% |
| Kwizera, R. 2021 | 110/286 | 33/88 | 1.04 [0.64, 1.71] | 0.0408 | 0.2515 | 14.4% |
| Saxena, P. 2021 | 61/205 | 103/338 | 0.97 [0.66, 1.41] | -0.0341 | 0.1931 | 14.9% |
| Subtotal (95% CI) |  |  | 2.36 [1.29, 4.31] |  |  | 100.0% |

| ***Penicillium* species** | **Sensitized / total** | | **OR and 95% CI** | **log [Odds Ratio]** | **SE** | **Weight** |
| --- | --- | --- | --- | --- | --- | --- |
|  | **Severe asthma** | **Non-severe asthma** |  |  |  |  |
| Ogawa, H. 2011 | 4/46 | 2/46 | 2.10 [0.36, 12.05] | 0.7397 | 0.8925 | 9.8% |
| Hayes, D. 2013 | Not reported | Not reported | 1.75 [0.53, 5.77] | 0.5624 | 0.6069 | 21.1% |
| Niedoszytko, M. 2007 | 7/44 | 7/61 | 1.46 [0.47, 4.51] | 0.3781 | 0.5756 | 23.5% |
| Tanaka, A. 2016 | Not reported | Not reported | 1.85 [0.83, 4.16] | 0.6178 | 0.4124 | 45.7% |
| Subtotal (95% CI) |  |  | 1.75 [1.02, 3.03] |  |  | 100.0% |

| ***Alternaria* species** | **Sensitized / total** | | **OR and 95% CI** | **log [Odds Ratio]** | **SE** | **Weight** |
| --- | --- | --- | --- | --- | --- | --- |
|  | **Severe asthma** | **Non-severe asthma** |  |  |  |  |
| Ogawa, H. 2011 | 2/46 | 1/46 | 2.05 [0.18, 23.38] | 0.7156 | 1.243 | 4.0% |
| Vincent, M. 2018 | 1/13 | 6/51 | 0.63 [0.07, 5.70] | -0.47 | 1.1279 | 4.8% |
| Beeh, K. M. 2001 | 2/22 | 6/90 | 1.40 [0.26, 7.46] | 0.3365 | 0.8536 | 8.4% |
| Hayes, D. 2013 | Not reported | Not reported | 1.70 [0.59, 4.91] | 0.5333 | 0.5394 | 21.0% |
| Niedoszytko, M. 2007 | 9/44 | 16/61 | 0.72 [0.29, 1.83] | -0.324 | 0.4737 | 27.2% |
| Tanaka, A. 2016 | Not reported | Not reported | 0.92 [0.40, 2.08] | -0.0888 | 0.4196 | 34.7% |
| Subtotal (95% CI) |  |  | 1.03 [0.63, 1.67] |  |  | 100.0% |

| ***Cladosporium* species** | **Sensitized / total** | | **OR and 95% CI** | **log [Odds Ratio]** | **SE** | **Weight** |
| --- | --- | --- | --- | --- | --- | --- |
|  | **Severe asthma** | **Non-severe asthma** |  |  |  |  |
| Gupta, A. 2015 | 3/58 | 1/42 | 2.24 [0.22, 22.29] | 0.8049 | 1.173 | 3.0% |
| Hayes, D. 2013 | Not reported | Not reported | 4.26 [1.07, 16.93] | 1.4504 | 0.7033 | 8.4% |
| Niedoszytko, M. 2007 | 9/44 | 6/61 | 2.36 [0.77, 7.20] | 0.8575 | 0.5697 | 12.8% |
| Tanaka, A. 2016 | Not reported | Not reported | 1.35 [0.55, 3.28] | 0.2968 | 0.4546 | 20.1% |
| Cazzoletti, L. 2010 | 37/393 | 23/734 | 3.21 [1.88, 5.49] | 1.1672 | 0.2734 | 55.6% |
| Subtotal (95% CI) |  |  | 2.63 [1.76, 3.92] |  |  | 100.0% |

| ***Helminthosporium* species** | **Sensitized / total** | | **OR and 95% CI** | **log [Odds Ratio]** | **SE** | **Weight** |
| --- | --- | --- | --- | --- | --- | --- |
|  | **Severe asthma** | **Non-severe asthma** |  |  |  |  |
| Gupta, A. 2015 | 2/58 | 0/42 | 3.76 [0.18, 80.39] | 1.3247 | 1.5624 | 4.8% |
| Hayes, D. 2013 | Not reported | Not reported | 1.35 [0.47, 3.89] | 0.3037 | 0.5376 | 40.4% |
| Niedoszytko, M. 2007 | 13/44 | 12/61 | 1.71 [0.69, 4.23] | 0.5379 | 0.4614 | 54.8% |
| Subtotal (95% CI) |  |  | 1.62 [0.83, 3.16] |  |  | 100.0% |

| ***Epicoccum* species** | **Sensitized / total** | | **OR and 95% CI** | **log [Odds Ratio]** | **SE** | **Weight** |
| --- | --- | --- | --- | --- | --- | --- |
|  | **Severe asthma** | **Non-severe asthma** |  |  |  |  |
| Hayes, D. 2013 | Not reported | Not reported | 1.57 [0.44, 5.68] | 0.4542 | 0.6541 | 38.7% |
| Niedoszytko, M. 2007 | 9/44 | 9/61 | 1.49 [0.54, 4.11] | 0.3959 | 0.5196 | 61.3% |
| Subtotal (95% CI) |  |  | 1.52 [0.68, 3.37] |  |  | 100.0% |

| ***Fusarium* species** | **Sensitized / total** | | **OR and 95% CI** | **log [Odds Ratio]** | **SE** | **Weight** |
| --- | --- | --- | --- | --- | --- | --- |
|  | **Severe asthma** | **Non-severe asthma** |  |  |  |  |
| Hayes, D. 2013 | Not reported | Not reported | 0.91 [0.24, 3.52] | -0.0889 | 0.6868 | 27.3% |
| Niedoszytko, M. 2007 | 16/44 | 18/61 | 1.37 [0.60, 3.11] | 0.3112 | 0.4207 | 72.7% |
| Subtotal (95% CI) |  |  | 1.22 [0.61, 2.47] |  |  | 100.0% |

| ***Candida* species** | **Sensitized / total** | | **OR and 95% CI** | **log [Odds Ratio]** | **SE** | **Weight** |
| --- | --- | --- | --- | --- | --- | --- |
|  | **Severe asthma** | **Non-severe asthma** |  |  |  |  |
| Gupta, A. 2015 | 2/58 | 0/42 | 3.76 [0.18, 80.39] | 1.3247 | 1.5624 | 3.8% |
| Niedoszytko, M. 2007 | 25/44 | 35/61 | 0.98 [0.45, 2.14] | -0.0228 | 0.3996 | 34.9% |
| Ogawa, H. 2011 | 13/46 | 4/46 | 4.14 [1.23, 13.87] | 1.4198 | 0.6173 | 19.5% |
| Tanaka, A. 2016 | Not reported | Not reported | 1.27 [0.66, 2.47] | 0.2425 | 0.3373 | 41.7% |
| Subtotal (95% CI) |  |  | 1.52 [0.82, 2.81] |  |  | 100.0% |

| ***Mucor* species** | **Sensitized / total** | | **OR and 95% CI** | **log [Odds Ratio]** | **SE** | **Weight** |
| --- | --- | --- | --- | --- | --- | --- |
|  | **Severe asthma** | **Non-severe asthma** |  |  |  |  |
| Gupta, A. 2015 | 0/58 | 5/42 | 0.06 [0.00, 1.08] | -2.8426 | 1.4918 | 8.9% |
| Niedoszytko, M. 2007 | 11/44 | 17/61 | 0.86 [0.36, 2.09] | -0.1476 | 0.4503 | 43.5% |
| Tanaka, A. 2016 | Not reported | Not reported | 1.23 [0.56, 2.67] | 0.2054 | 0.3963 | 47.7% |
| Subtotal (95% CI) |  |  | 0.80 [0.32, 2.04] |  |  | 100.0% |

| ***Trichophyton* species** | **Sensitized / total** | | **OR and 95% CI** | **log [Odds Ratio]** | **SE** | **Weight** |
| --- | --- | --- | --- | --- | --- | --- |
|  | **Severe asthma** | **Non-severe asthma** |  |  |  |  |
| Ogawa, H. 2011 | 5/46 | 4/46 | 1.28 [0.32, 5.11] | 0.2472 | 0.7058 | 23.4% |
| Tanaka, A. 2016 | Not reported | Not reported | 1.40 [0.65, 3.01] | 0.3365 | 0.3906 | 76.6% |
| Subtotal (95% CI) |  |  | 1.37 [0.70, 2.68] |  |  | 100.0% |

- 1. Table S4: Calculation process of Aspergillus species subgroup analysis

We conducted a subgroup analysis of the previously calculated odds ratio (OR) values for the genus *Aspergillus* based on the characteristics of the original study populations. We combined these values using a random-effects model. All OR values and their 95% confidence intervals (CIs) were logarithmically transformed through the built-in calculator of RevMan, and the inverse variance method was applied for weighting. The Generic Inverse Variance method, by default, adopted the DerSimonian-Laird estimator. This estimator calculates the between-study heterogeneity variance (*Tau²*) and dynamically adjusts the weights of the effect sizes of individual studies to reflect the differences among studies, thereby enabling a robust estimation of the pooled effect size.

| **Average age < 40 years** | **Sensitized / total** | | **OR and 95% CI** | **log [Odds Ratio]** | **SE** | **Weight** |
| --- | --- | --- | --- | --- | --- | --- |
|  | **Severe asthma** | **Non-severe asthma** |  |  |  |  |
| Chopra, V. 2017 | 90/93 | 116/189 | 18.88 [5.76, 61.87] | 2.9381 | 0.6056 | 14.3% |
| Hayes, D. 2013 | Not reported | Not reported | 1.33 [0.43, 4.17] | 0.2889 | 0.5806 | 14.7% |
| Kwizera, R. 2021 | 110/286 | 33/88 | 1.04 [0.64, 1.71] | 0.0408 | 0.2515 | 19.4% |
| Niedoszytko, M. 2007 | 11/44 | 10/61 | 1.70 [0.65, 4.45] | 0.5306 | 0.4907 | 16.0% |
| Saxena, P. 2021 | 61/205 | 103/338 | 0.97 [0.66, 1.41] | -0.0341 | 0.1931 | 20.1% |
| Yeğit, O. O. 2023 | 23/33 | 10/44 | 7.82 [2.81, 21.77] | 2.0567 | 0.5224 | 15.5% |
| Subtotal (95% CI) |  |  | 2.38 [1.08, 5.26] |  |  | 100.0% |

| **Average age > 40 years** | **Sensitized / total** | | **OR and 95% CI** | **log [Odds Ratio]** | **SE** | **Weight** |
| --- | --- | --- | --- | --- | --- | --- |
|  | **Severe asthma** | **Non-severe asthma** |  |  |  |  |
| Ogawa, H. 2011 | 4/46 | 3/46 | 1.37 [0.29, 6.47] | 0.3112 | 0.794 | 14.4% |
| Tanaka, A. 2016 | Not reported | Not reported | 2.20 [1.10, 4.44] | 0.7907 | 0.3571 | 71.2% |
| Vincent, M. 2018 | 4/13 | 4/51 | 5.22 [1.10, 24.82] | 1.6529 | 0.7952 | 14.4% |
| Subtotal (95% CI) |  |  | 2.33 [1.29, 4.20] |  |  | 100.0% |

| **M/F > 1** | **Sensitized / total** | | **OR and 95% CI** | **log [Odds Ratio]** | **SE** | **Weight** |
| --- | --- | --- | --- | --- | --- | --- |
|  | **Severe asthma** | **Non-severe asthma** |  |  |  |  |
| Ogawa, H. 2011 | 4/46 | 3/46 | 1.37 [0.29, 6.47] | 0.3112 | 0.794 | 50.1% |
| Vincent, M. 2018 | 4/13 | 4/51 | 5.22 [1.10, 24.82] | 1.6529 | 0.7952 | 49.9% |
| Subtotal (95% CI) |  |  | 2.67 [0.72, 9.94] |  |  | 100.0% |

| **M/F < 1** | **Sensitized / total** | | **OR and 95% CI** | **log [Odds Ratio]** | **SE** | **Weight** |
| --- | --- | --- | --- | --- | --- | --- |
|  | **Severe asthma** | **Non-severe asthma** |  |  |  |  |
| Chopra, V. 2017 | 90/93 | 116/189 | 18.88 [5.76, 61.87] | 2.9381 | 0.6056 | 11.7% |
| Hayes, D. 2013 | Not reported | Not reported | 1.33 [0.43, 4.17] | 0.2889 | 0.5806 | 12.1% |
| Kwizera, R. 2021 | 110/286 | 33/88 | 1.04 [0.64, 1.71] | 0.0408 | 0.2515 | 16.8% |
| Niedoszytko, M. 2007 | 11/44 | 10/61 | 1.70 [0.65, 4.45] | 0.5306 | 0.4907 | 13.4% |
| Saxena, P. 2021 | 61/205 | 103/338 | 0.97 [0.66, 1.41] | -0.0341 | 0.1931 | 17.5% |
| Tanaka, A. 2016 | Not reported | Not reported | 2.20 [1.10, 4.44] | 0.7907 | 0.3571 | 15.4% |
| Yeğit, O. O. 2023 | 23/33 | 10/44 | 7.82 [2.81, 21.77] | 2.0567 | 0.5224 | 13.0% |
| Subtotal (95% CI) |  |  | 2.32 [1.18, 4.54] |  |  | 100.0% |

| ***Aspergillus fumigatus*** | **Sensitized / total** | | **OR and 95% CI** | **log [Odds Ratio]** | **SE** | **Weight** |
| --- | --- | --- | --- | --- | --- | --- |
|  | **Severe asthma** | **Non-severe asthma** |  |  |  |  |
| Chopra, V. 2017 | 90/93 | 116/189 | 18.88 [5.76, 61.87] | 2.9381 | 0.6056 | 14.5% |
| Kwizera, R. 2021 | 110/286 | 33/88 | 1.04 [0.64, 1.71] | 0.0408 | 0.2515 | 19.5% |
| Saxena, P. 2021 | 61/205 | 103/338 | 0.97 [0.66, 1.41] | -0.0341 | 0.1931 | 20.1% |
| Tanaka, A. 2016 | Not reported | Not reported | 2.20 [1.10, 4.44] | 0.7907 | 0.3571 | 18.2% |
| Vincent, M. 2018 | 4/13 | 4/51 | 5.22 [1.10, 24.82] | 1.6529 | 0.7952 | 11.9% |
| Yeğit, O. O. 2023 | 23/33 | 10/44 | 7.82 [2.81, 21.77] | 2.0567 | 0.5224 | 15.8% |
| Subtotal (95% CI) |  |  | 2.98 [1.32, 6.75] |  |  | 100.0% |

| **Average age > 40 years (*Aspergillus fumigatus*)** | **Sensitized / total** | | **OR and 95% CI** | **log [Odds Ratio]** | **SE** | **Weight** |
| --- | --- | --- | --- | --- | --- | --- |
|  | **Severe asthma** | **Non-severe asthma** |  |  |  |  |
| Tanaka, A. 2016 | Not reported | Not reported | 2.20 [1.10, 4.44] | 0.7907 | 0.3571 | 83.2% |
| Vincent, M. 2018 | 4/13 | 4/51 | 5.22 [1.10, 24.82] | 1.6529 | 0.7952 | 16.8% |
| Subtotal (95% CI) |  |  | 2.55 [1.35, 4.83] |  |  | 100.0% |

| **Average age < 40 years (*Aspergillus fumigatus*)** | **Sensitized / total** | | **OR and 95% CI** | **log [Odds Ratio]** | **SE** | **Weight** |
| --- | --- | --- | --- | --- | --- | --- |
|  | **Severe asthma** | **Non-severe asthma** |  |  |  |  |
| Chopra, V. 2017 | 90/93 | 116/189 | 18.88 [5.76, 61.87] | 2.9381 | 0.6056 | 21.6% |
| Kwizera, R. 2021 | 110/286 | 33/88 | 1.04 [0.64, 1.71] | 0.0408 | 0.2515 | 27.3% |
| Saxena, P. 2021 | 61/205 | 103/338 | 0.97 [0.66, 1.41] | -0.0341 | 0.1931 | 28.0% |
| Yeğit, O. O. 2023 | 23/33 | 10/44 | 7.82 [2.81, 21.77] | 2.0567 | 0.5224 | 23.1% |
| Subtotal (95% CI) |  |  | 3.04 [1.01, 9.12] |  |  | 100.0% |

| **M/F < 1 (*Aspergillus fumigatus*)** | **Sensitized / total** | | **OR and 95% CI** | **log [Odds Ratio]** | **SE** | **Weight** |
| --- | --- | --- | --- | --- | --- | --- |
|  | **Severe asthma** | **Non-severe asthma** |  |  |  |  |
| Chopra, V. 2017 | 90/93 | 116/189 | 18.88 [5.76, 61.87] | 2.9381 | 0.6056 | 16.5% |
| Kwizera, R. 2021 | 110/286 | 33/88 | 1.04 [0.64, 1.71] | 0.0408 | 0.2515 | 22.1% |
| Saxena, P. 2021 | 61/205 | 103/338 | 0.97 [0.66, 1.41] | -0.0341 | 0.1931 | 22.8% |
| Tanaka, A. 2016 | Not reported | Not reported | 2.20 [1.10, 4.44] | 0.7907 | 0.3571 | 20.6% |
| Yeğit, O. O. 2023 | 23/33 | 10/44 | 7.82 [2.81, 21.77] | 2.0567 | 0.5224 | 17.9% |
| Subtotal (95% CI) |  |  | 2.77 [1.16, 6.62] |  |  | 100.0% |

| **Only SPT** | **Sensitized / total** | | **OR and 95% CI** | **log [Odds Ratio]** | **SE** | **Weight** |
| --- | --- | --- | --- | --- | --- | --- |
|  | **Severe asthma** | **Non-severe asthma** |  |  |  |  |
| Chopra, V. 2017 | 90/93 | 116/189 | 18.88 [5.76, 61.87] | 2.9381 | 0.6056 | 16.1% |
| Hayes, D. 2013 | Not reported | Not reported | 1.33 [0.43, 4.17] | 0.2889 | 0.5806 | 16.6% |
| Kwizera, R. 2021 | 110/286 | 33/88 | 1.04 [0.64, 1.71] | 0.0408 | 0.2515 | 23.9% |
| Niedoszytko, M. 2007 | 11/44 | 10/61 | 1.70 [0.65, 4.45] | 0.5306 | 0.4907 | 18.6% |
| Saxena, P. 2021 | 61/205 | 103/338 | 0.97 [0.66, 1.41] | -0.0341 | 0.1931 | 24.9% |
| Subtotal (95% CI) |  |  | 1.86 [0.87, 3.97] |  |  | 100.0% |

| **Not Only SPT** | **Sensitized / total** | | **OR and 95% CI** | **log [Odds Ratio]** | **SE** | **Weight** |
| --- | --- | --- | --- | --- | --- | --- |
|  | **Severe asthma** | **Non-severe asthma** |  |  |  |  |
| Ogawa, H. 2011 | 4/46 | 3/46 | 1.37 [0.29, 6.47] | 0.3112 | 0.794 | 17.0% |
| Tanaka, A. 2016 | Not reported | Not reported | 2.20 [1.10, 4.44] | 0.7907 | 0.3571 | 38.1% |
| Vincent, M. 2018 | 4/13 | 4/51 | 5.22 [1.10, 24.82] | 1.6529 | 0.7952 | 16.9% |
| Yeğit, O. O. 2023 | 23/33 | 10/44 | 7.82 [2.81, 21.77] | 2.0567 | 0.5224 | 28.0% |
| Subtotal (95% CI) |  |  | 3.35 [1.56, 7.23] |  |  | 100.0% |

- 1. Figure S1: Funnel plots for Sensitization to Aspergillus spp. and severe asthma (A); Sensitization to Penicillium spp. and severe asthma (B);Sensitization to Cladosporium spp. and severe asthma (C); Sensitization to Alternaria spp. and severe asthma (D); Sensitization to Candida spp. and severe asthma (E)


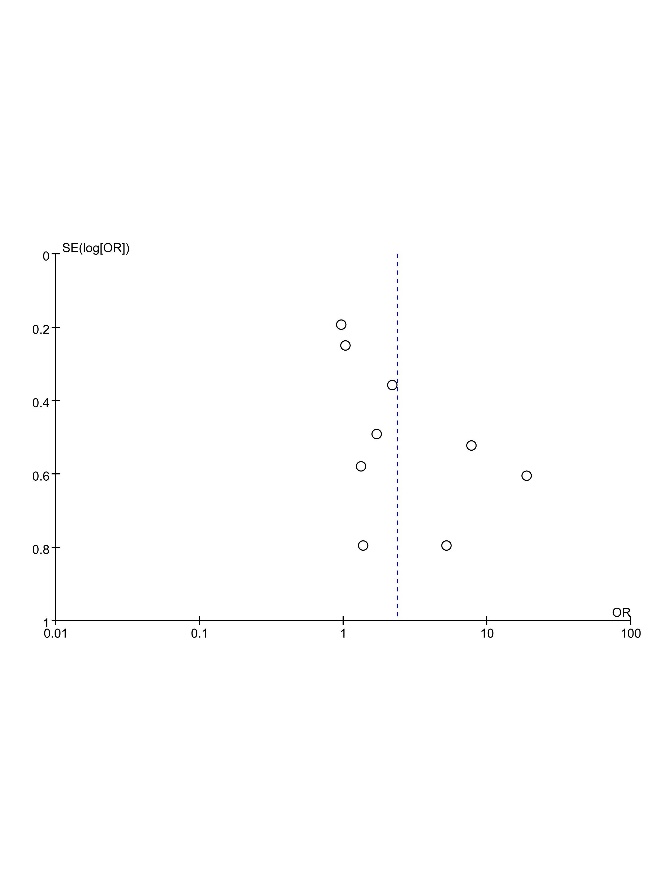

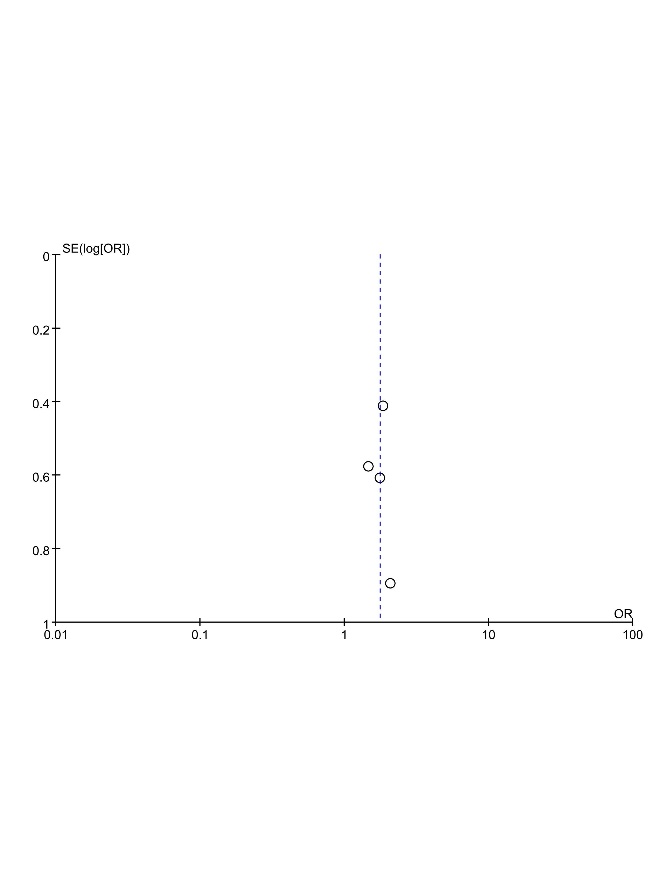


1. (B)


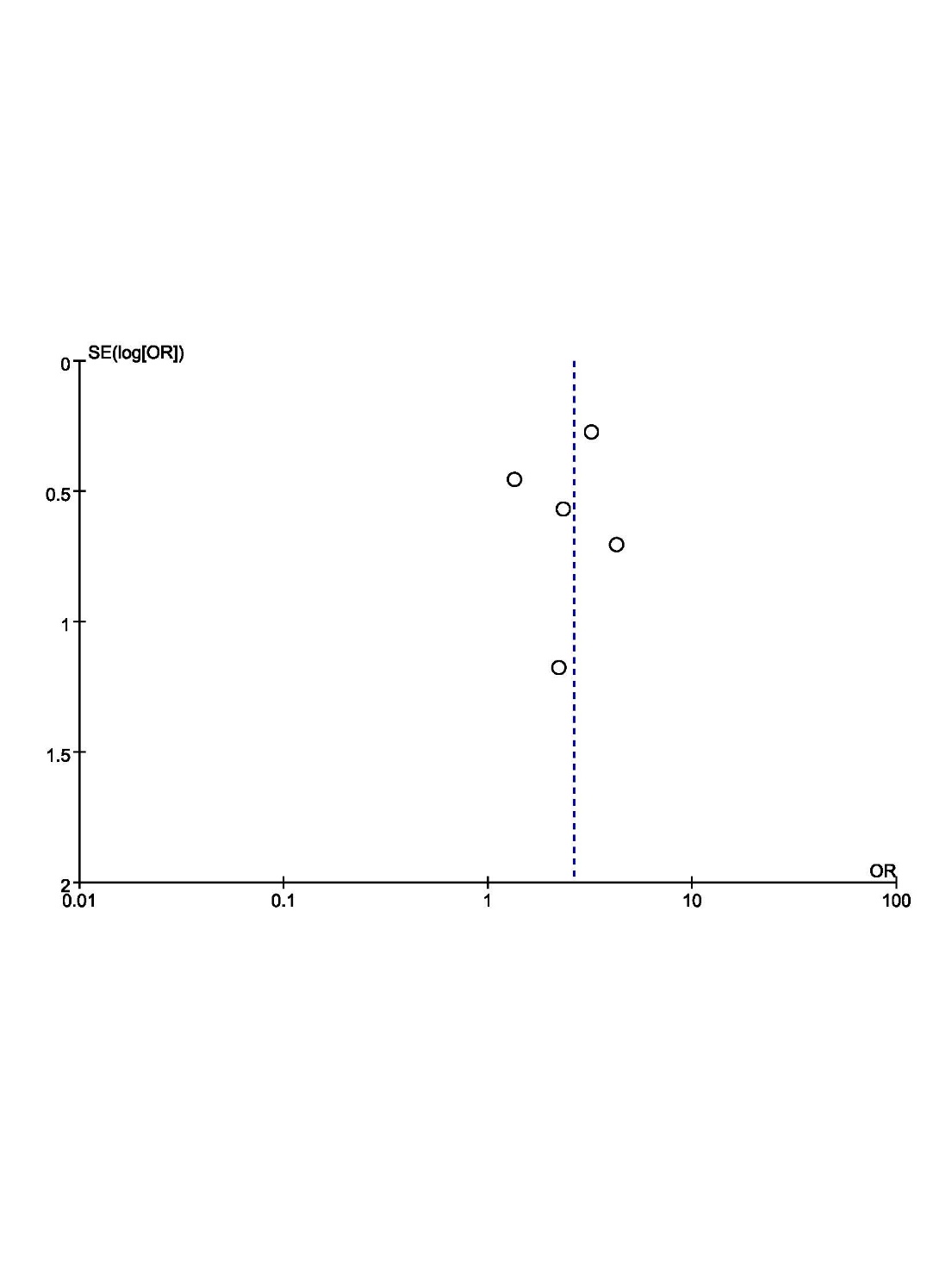

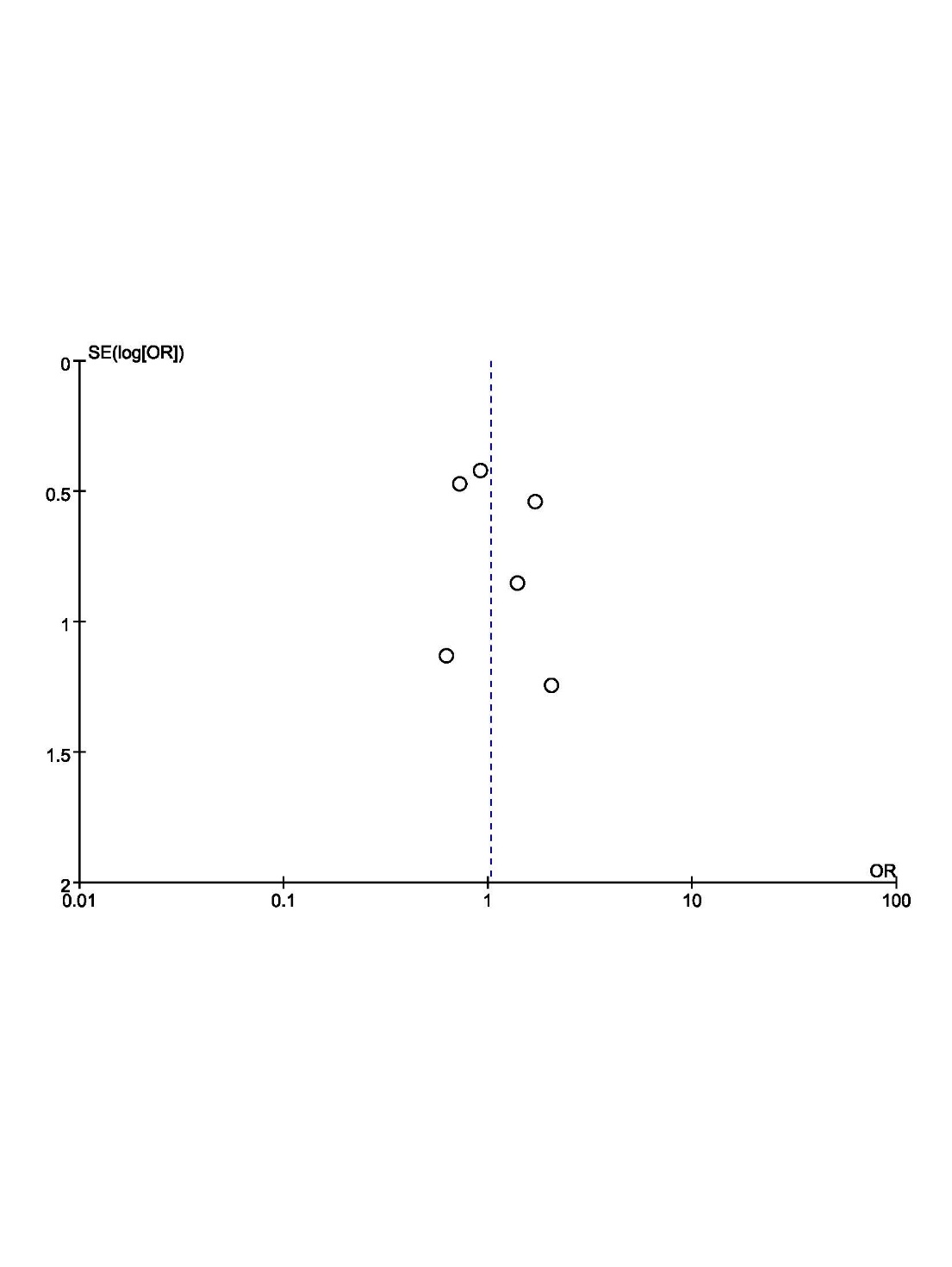


**(C)**  **(D)**


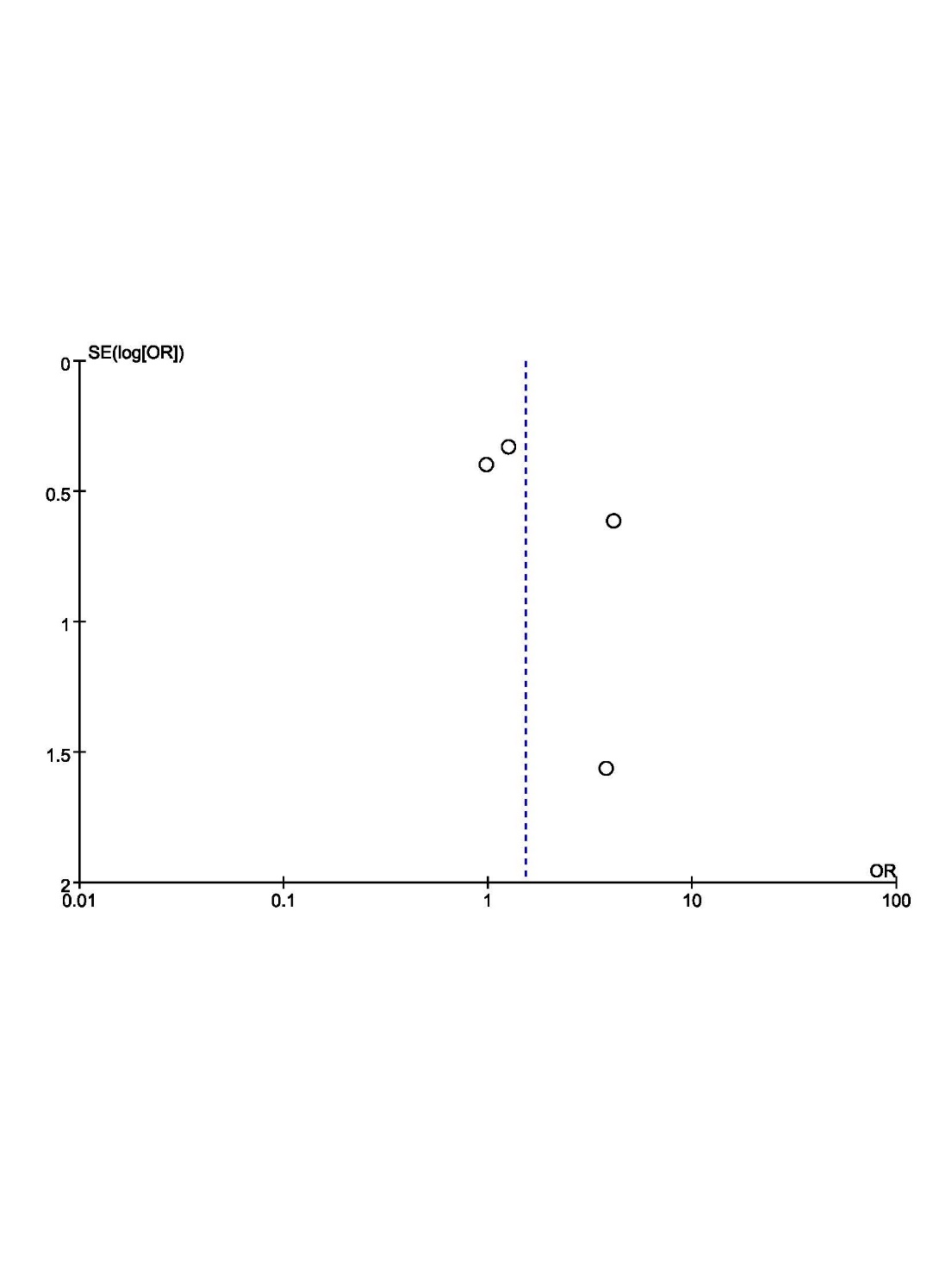


**(E)**
